# Equity impact of interventions to promote physical activity in older adults: protocol for a systematic review

**DOI:** 10.1186/s13643-016-0194-8

**Published:** 2016-02-01

**Authors:** Gesa Lehne, Gabriele Bolte

**Affiliations:** Department of Social Epidemiology, Institute for Public Health and Nursing Research, University of Bremen, Bremen, Germany; Health Sciences Bremen, University of Bremen, Bremen, Germany

**Keywords:** Physical activity, Social inequalities, Interventions, Evaluation, Older adults

## Abstract

**Background:**

Public health strategies to promote physical activity among older adults are increasingly being implemented. However, it is not known whether these interventions are equally effective among all social groups of the older adult population. The objectives of the proposed systematic review are to (1) describe the extent to which effects on social inequalities are considered in studies evaluating the effectiveness of interventions to promote physical activity among older adults, (2) describe the methods used for measuring these effects, and (3) assess the implications of the equity related findings for health promotion research and practice.

**Methods/design:**

MEDLINE, PsycINFO, CINAHL, CENTRAL, Physical Education Index, Social Science Citation Index, ASSIA, Sociological Abstracts, and IBSS databases as well as the German language journal Prävention und Gesundheitsförderung will be searched to identify experimental or observational quantitative studies evaluating the effects of interventions on self-reported or objectively measured physical activity among the general population of older adults (≥50 years). English or German language peer-reviewed journal articles published since 2005 will be included. Data on whether and how several social factors are considered for both the description of baseline characteristics of participants and for measuring intervention effectiveness will be extracted. The quality of studies will be assessed using the Effective Public Health Practice Project Quality Assessment Tool for Quantitative Studies. Results will be presented in a narrative synthesis. If feasible, harvest plots will be used to synthesize evidence about how intervention effects vary between different social groups.

**Discussion:**

This systematic review will provide evidence on what is known about the effects of interventions on social inequalities in physical activity among older adults, which is a prerequisite for the prioritization of those interventions most likely to be effective across all social groups of this target population. Therefore, the results of this review will be of major interest to researchers, policy makers, and practitioners in the area of physical activity promotion for older adults.

**Systematic review registration:**

This protocol has been registered with the PROSPERO international prospective register of systematic reviews (PROSPERO 2015 CRD42015025066).

**Electronic supplementary material:**

The online version of this article (doi:10.1186/s13643-016-0194-8) contains supplementary material, which is available to authorized users.

## Background

Due to various beneficial effects on health and well-being, physical activity is seen as one of the most important contributors to healthy aging [1]. For example, considerable evidence suggests that physical activity plays an important role in the primary and secondary prevention of major non-communicable diseases (e.g., cardiovascular diseases, type 2 diabetes mellitus, obesity, cancer, depression, chronic respiratory diseases, dementia, osteoporosis) [2–4]. Although the benefits of regular physical activity have also been shown to be effective among older adults [5–7], epidemiological studies suggest that physical activity level tends to decline with increasing age [8–10].

There is also evidence indicating that physical activity is less prevalent among socially disadvantaged population groups [11–14]. In this context, social inequalities may refer to a variety of socioeconomic, sociocultural, and sociogeographical aspects [11] which have been summarized within the PROGRESS-Plus framework proposed by the Campbell and Cochrane Equity Methods Group [15]. The acronym PROGRESS represents the following eight dimensions across which inequalities may exist: *P*lace of residence, *R*ace/ethnicity/culture, *O*ccupation, *G*ender/sex, *R*eligion, *E*ducation, *S*ocioeconomic status (SES), and *S*ocial capital [16]. “Plus” considers other characteristics of populations which may be associated with social disadvantage (e.g., age or disability) [17]. With regard to older adults and physical activity, low physical activity has been shown to be associated with female sex, low SES, living in a deprived residential area, and a lack of social support [9, 10, 18–20].

Ideally, health promotion and prevention measures should reach the whole social spectrum of a population and thus not increase social inequalities between socially advantaged and disadvantaged groups. However, there is a growing body of evidence suggesting that preventive interventions, even if they are successful at improving health behaviors or health outcomes across the population, may actually worsen inequalities between different social groups [21–23]. According to Lorenc and colleagues, these “intervention-generated inequalities” (IGIs) are more likely to occur among interventions focusing on individual behavior changes (downstream interventions) compared to interventions focusing on social or policy changes (upstream interventions) [24]. For example, using systematic review methods, Hill and colleagues have shown that increased tobacco price has the potential to reduce smoking-related inequalities in health among adults, whereas non-targeted smoking cessation programs seem to increase inequalities [25]. The issue of IGIs has also been discussed in systematic reviews of obesity prevention interventions among adults [26, 27] and children [28], interventions to improve healthy eating [29] and school-based cognitive-behavioral [30], and health behavior interventions [31].

In the area of physical activity promotion, Humphreys and Ogilvie conducted a pilot study on how effects on social inequalities have been reported in systematic reviews and primary studies of the effects of environmental and policy interventions to promote physical activity [32]. The authors found that only few systematic reviews tended to synthesize intervention effects on social inequalities, although relevant information (e.g., on participants’ baseline characteristics, adjusted associations, subgroup intervention effects, or interaction effects) was often provided within the primary studies included in the reviews.

### Objectives

The objectives of the proposed systematic review are to (1) describe the extent to which effects on social inequalities are considered in quantitative experimental or observational studies evaluating the effectiveness of interventions to promote physical activity among the general population of older adults (≥50 years), (2) describe the methods used for measuring these effects, and (3) assess the implications of the equity-related findings for health promotion research and practice.

## Methods/design

### Protocol and registration

This protocol has been registered with the PROSPERO international prospective register of systematic reviews (registration number: CRD42015025066) and was reported adhering to the Preferred Reporting Items for Systematic Review and Meta-Analysis Protocols (PRISMA-P) 2015 statement [33, 34] (see Additional file 1). The final review will be reported following the Preferred Reporting Items for Systematic Review and Meta-Analyses (PRISMA) statement and the equity extension of the PRISMA statement, PRISMA-E 2012 [35–38]. Important protocol amendments will be documented and published with the results of the review.

### Study selection criteria

#### Types of studies

This systematic review will include published peer-reviewed journal articles from 2005 to July 2015, written in English or German language. Randomized controlled trials (RCTs), which are often considered the gold standard when assessing the effectiveness of interventions, are likely to be less frequently conducted in the field of public health and health promotion [39]. Especially for environmental or policy level interventions, RCTs are rarely available [40]. Therefore, the review will include all types of quantitative experimental as well as observational study designs (with or without concurrent control group) which evaluated the effects of interventions on physical activity among older adults, such as RCTs, cluster randomized controlled trials (cluster RCTs), non-randomized controlled trials (NRCTs), controlled before-and-after (CBA) studies, cohort studies, interrupted-time-series (ITS) studies, or before-and-after (BA) studies. Studies using a cross-sectional design, for example, for evaluating the effects of an intervention in the outdoor environment by comparing the intervention area with a reference area, will also be eligible.

#### Types of participants

Studies will be included if the study population comprises people aged 50 years and over. Only studies on interventions that potentially address everyone across the social spectrum (universal interventions) will be included.

Thus, studies in which the study population is restricted to particular social groups of older adults (e.g., studies targeted at women or men or at low SES population groups only) will be excluded. Studies whose study participants are restricted with regard to actual physical activity behavior (e.g., study population is described as “inactive", “insufficiently active", “sedentary", “physically active”), functional status (e.g., study population is described as “sarcopenic", “home-bound", “fall-prone", “functionally impaired", “frail”) or specific underlying medical conditions (e.g., dementia, cancer, depression, multiple sclerosis, diabetes) will also be excluded. Furthermore, studies whose study populations are restricted to overweight or obese individuals as well as studies focused on participants receiving nursing or rehabilitation care will not be included.

Studies in which participants met one or more of the abovementioned conditions, but have not been recruited specifically due to these conditions, will be included. Additionally, eligible studies have to report baseline characteristics of participants stratified by at least one social factor according to the PROGRESS-Plus framework. Since only studies focused on older adults will be included in the review, age alone will not be considered as sufficient to fulfill this inclusion criterion.

#### Types of interventions

Studies will be included if they evaluate the effects of interventions on physical activity behavior among older adults either as the study’s main objective or within a comprehensive intervention design with multiple intervention components. Eligible interventions may operate at various levels (i.e., individual, community, or societal level interventions) and may be delivered in a variety of modes (e.g., face-to-face, phone call, online, smartphone, changes to the built environment) with various frequency, duration, or intensity characteristics. No restrictions on intervention and follow-up duration will be applied.

#### Types of comparators

The review will include quantitative study designs with and without concurrent control groups. Therefore, where applicable, controls may be active (i.e., receiving an alternative intervention approach) or passive (e.g., receiving no intervention, usual care, or waitlist control group). Studies which compare the effects of an intervention between residents of two areas, in one of which an intervention was implemented, will also be eligible.

#### Types of outcome measures

To be included in the review, studies have to report changes in physical activity, either assessed objectively (e.g., by pedometers or accelerometers) or subjectively (e.g., by questionnaires). Studies only measuring changes in psychological outcomes (e.g., intentions, self-efficacy, knowledge, attitudes) regarding physical activity instead of changes in physical activity behavior itself will be excluded. Furthermore, studies in which effects on physical activity are only assessed as changes in physical function measures (e.g., muscle function, grip strength, flexibility, gait speed, postural control) will also be excluded.

### Search strategy

#### Electronic searches

To identify relevant studies, the following electronic databases will be searched:

MEDLINE (via PubMed), PsycINFO (via Ovid), Cumulative Index to Nursing and Allied Health Literature (CINAHL) (via EBSCO Host), Cochrane Register of Controlled trials (CENTRAL) (via Cochrane Library), Physical Education Index (via ProQuest), Social Science Citation Index (SSCI) (via Web of Science), Applied Social Sciences Index and Abstracts (ASSIA) (via ProQuest), Sociological Abstracts (via ProQuest), and International Bibliography of the Social Sciences (IBSS) (via ProQuest). Restrictions on English or German language as well as on articles published between 2005 and present will be applied. The search strategy will comprise searching text words in titles and abstracts, where applicable. Search terms related to (1) physical activity, (2) interventions, (3) intervention effects, and (4) older adults will be established by examining previous systematic reviews in the area of interest and discussion between authors. The sensitivity of the search strategy will be tested by examining whether it retrieves several key articles identified in preliminary searches. A sample search strategy for MEDLINE designed in PubMed is shown in Additional file 2.

#### Searching other resources

The references cited of all articles meeting the inclusion criteria will be screened to capture any relevant publications missed by the electronic searches. To increase the chance of finding relevant articles published in German language not included in the electronic databases, the German language journal Prävention und Gesundheitsförderung (Springer Medizin; volume 1, issue 1, January 2006—volume 10, issue 2, May 2015) will be manually searched.

#### Study selection

An EndNote (ENDNOTE X7.1, Thomson Reuters) database will be created to store all citations retrieved by the nine electronic literature databases. Using EndNote’s auto-deduplication function, duplicate citations will be removed. Since auto-deduplication is thought to be only partially successful [41], the remaining duplicates will be identified by hand-searching techniques. To do this, references will be alphabetically ordered according to the first authors’ names and thereafter according to their titles.

### First stage screening

After removing duplicates, first, all remaining titles and abstracts will initially be screened by two reviewers independently. References will be designated as either “not eligible” or “potentially eligible". To differentiate between not and potentially eligible, the following criteria regarding study characteristics, study participants, and interventions will be applied:

- *Study characteristics*: “Potentially eligible” references have to be studies published as an electronic journal article between 2005 and present, and written in English or German language.
- *Study participants*: To be “potentially eligible", the title or abstract must clearly state that the study population comprises older adults or people aged 50 years and over, respectively. Since there is no common definition of “older adults” in the literature, non-specific descriptions, such as “middle aged and older adults", will also be accepted at this stage of screening. References whose titles or abstracts clearly state that the study population is restricted with regard to actual physical activity behavior, functional status, medical conditions, or weight status will be deemed “not eligible".
- *Interventions*: To be “potentially eligible", the title or abstract must be clear that the study reports the effects of an intervention on physical activity behavior. Changes in physical function measures or psychological outcomes only will not be accepted.

Second, both reviewers will compare their decisions and discuss all references generating a disagreement. If necessary, a third reviewer will be consulted. Before consensus will be achieved, inter-rater reliability between the two reviewers will be assessed using a Cohen’s kappa statistic [42].

### Second stage screening

Full texts of potentially eligible references will be retrieved and assessed for final inclusion by one reviewer with a random sample checked by a second reviewer. Following PRISMA guidelines [35], a flow diagram will be created to illustrate the study selection process, with explanations provided for those studies excluded in the second stage screening process.

### Data extraction and management

Data extraction will be conducted by one reviewer and checked by a second reviewer using a pre-designed and pilot-tested data extraction form. It is anticipated that many studies considered social factors described by PROGRESS-Plus only for the description of baseline characteristics of participants. Therefore, a two-stage approach for data extraction will be applied. The following information will be extracted from all included studies (stage one):

- Bibliographic details (first author, year of study, publication language, country of study)
- Study design and study aim(s)
- Eligible participants (inclusion and exclusion criteria)
- Intervention details (intervention aim(s), level of intervention and setting, intervention content, control/comparison, intervention delivery, intervention duration, follow-up duration)
- Physical activity outcome, outcome measurement, and time points of measurements
- Number of participants and PROGRESS-Plus characteristics reported at baseline
- Intervention effects on physical activity
- Whether study considered PROGRESS-Plus dimensions for measuring intervention effects on physical activity outcome

For studies which examined differential intervention effects by PROGRESS-Plus characteristics (i.e., analyzing subgroup intervention effects or interaction effects by PROGRESS-Plus characteristics), an expanded data collection form will be applied (stage 2). This will include more detailed information on study methods (e.g., method of recruitment of participants, existence of study protocol), intervention characteristics (e.g., theoretical underpinning), and study results (e.g., number and reason for and sociodemographic or socioeconomic differences of withdrawals and dropouts) as well as on the statistical methods used for examining differential intervention effects. Furthermore, information on the ways in which inequalities are expressed (absolute versus relative terms) will be extracted.

### Risk of bias assessment of included studies

Quality appraisal of all included studies which examined differential intervention effects by PROGRESS-Plus characteristics will be performed by two reviewers independently. Any disagreements will be resolved through discussion and, if necessary, a third reviewer will be consulted. Quality will be assessed using the Effective Public Health Practice Project (EPHPP) “Quality Assessment Tool for Quantitative Studies” [43]. The EPHPP tool is recommended by the Cochrane Public Health Group as it has been shown to be applicable across a variety of intervention study designs [44]. The tool covers the following domains: (A) selection bias, (B) study design, (C) confounders, (D) blinding, (E) data collection method, and (F) withdrawals and dropouts. Intervention integrity and analysis methods are also appraised.

### Data synthesis

This systematic review will use a very broad search strategy to capture the entire evidence base on the effects of universal interventions on social inequalities in physical activity among older adults. It is anticipated that this strategy will result in a diverse range of research methods (e.g., regarding study design, intervention characteristics, setting, participant characteristics, outcome measures). Hence, using meta-analysis to integrate and summarize the included studies is unlikely to be appropriate. Instead, a narrative synthesis of results will be conducted, including tables and figures of information relevant for the primary objectives of the review.

To assess the extent to which studies considered effects of interventions on social inequalities, two levels of analysis will be applied: At level 1, we will present data on whether the studies considered various dimensions of social inequalities described by PROGRESS-Plus for the description of baseline characteristics of study participants. At level 2, we will describe whether the studies considered PROGRESS-Plus dimensions for measuring intervention effects and which methods they used (e.g., consideration as confounders by adjusting in multivariate analyses, as effect modifiers in stratified analyses or using interaction terms in multivariate analyses). If feasible, studies which examined differential intervention effects by PROGRESS-Plus characteristics will be synthesized using harvest plots [45]. In addition, the ways in which inequalities are expressed, that is, absolute versus relative inequalities, will be examined, since the decision about whether to measure inequalities in relative or absolute terms may influence their interpretation [46].

## Discussion

To our knowledge, this systematic review will be the first to describe the extent to which and the ways in which effects on social inequalities are considered in studies evaluating the effectiveness of interventions to promote physical activity among the general population of older adults. It will further give an impression of how many universal intervention studies provide data on various social factors but do not examine, or at least do not report having examined possible effects on social inequalities. We anticipate that the results of this systematic review will be of interest to multiple stakeholders, including researchers, policy makers, and practitioners in the area of physical activity promotion for older adults. This systematic review will focus on universal interventions that potentially address everyone across the social spectrum of the older adult population. Interventions designed to specifically target certain socially disadvantaged population groups will not be included. The effectiveness of these targeted interventions in terms of equity impacts should be analyzed in a separate review.

The dissemination plan of findings of this review includes the scientific publication in an academic journal as well as the presentation at relevant scientific conferences. Furthermore, findings will be disseminated through research and university networks.

### Ethics approval and consent to participate

Not applicable.

### Consent for publication

Not applicable.

## Additional files

Additional file 1:
**PRISMA-P (Preferred Reporting Items for Systematic Review and Meta-Analysis Protocols) 2015 checklist: recommended items to include in a systematic review protocol.** This file provides a completed PRISMA-P 2015 checklist. (DOC 82 kb) Additional file 2:
**Sample search string for PubMed MEDLINE.** This file contains a sample search string for PubMed MEDLINE. (DOCX 14 kb)

## Abbreviations

ASSIA: Applied Social Science Index and Abstracts
BA: before-and-after
CBA: controlled before-and-after
CENTRAL: Cochrane Register of Controlled Trials
CINAHL: Cumulative Index to Nursing and Allied Health Literature
CRD: Centre for Reviews and Dissemination
EPHPP: Effective Public Health Practice Project
IBSS: International Bibliography of the Social Sciences
IGIs: intervention-generated inequalities
ITS: interrupted-time-series
NRCTs: non-randomized controlled trials
PRISMA: Preferred Reporting Items for Systematic Reviews and Meta-Analyses
PRISMA-E: PRISMA-Equity 2012 Extension, Reporting Guidelines for Systematic Reviews with a Focus on Health Equity
PRISMA-P: Preferred Reporting Items for Systematic Review and Meta-analysis Protocols
PROGRESS-Plus: Place of residence, Race/ethnicity/culture, Occupation, Gender/sex, Religion, Education, Socioeconomic status, Social capital. “Plus” considers other categories that may impact on health equity (e.g., age or disability)
PROSPERO: International Prospective Register of Systematic Reviews
RCTs: randomized controlled trials
SES: socioeconomic status
SSCI: Social Science Citation Index
