# Supplementary material for: Equity impact of interventions to promote physical activity in older adults: protocol for a systematic review
Source: Syst Rev. 2016 Feb 1;5:17. doi: 10.1186/s13643-016-0194-8 (PMC4736241; doi:10.1186/s13643-016-0194-8)
Supplement: Additional file 2: — Sample search string for PubMed MEDLINE. This file contains a sample search string for PubMed MEDLINE. (DOCX 14 kb) [file 13643_2016_194_MOESM2_ESM.docx]

**Additional file 2**

**Sample search string for PubMed MEDLINE**

Limits: publication date: last 10 years, publication languages: English, German

Truncation symbol: * = all possible word endings included

((physical activi*[Title/Abstract] OR exercis*[Title/Abstract] OR sport*[Title/Abstract] OR walk*[Title/Abstract] OR active transport*[Title/Abstract] OR active commut*[Title/Abstract] OR sedentary behavio*[Title/Abstract] OR physical inactivi*[Title/Abstract]) AND (intervention*[Title/Abstract] OR program*[Title/Abstract] OR policy[Title/Abstract] OR policies[Title/Abstract] OR project*[Title/Abstract] OR campaign*[Title/Abstract] OR strateg*[Title/Abstract]) AND (evaluat*[Title/Abstract] OR efficacy[Title/Abstract] OR effect*[Title/Abstract] OR affect*[Title/Abstract] OR impact*[Title/Abstract] OR trial*[Title/Abstract]) AND (elder*[Title/Abstract] OR old people[Title/Abstract] OR older people[Title/Abstract] OR old adult*[Title/Abstract] OR old person*[Title/Abstract] OR older adult*[Title/Abstract] OR older person*[Title/Abstract] OR senior*[Title/Abstract] OR aging person*[Title/Abstract] OR ageing person*[Title/Abstract] OR aging adult*[Title/Abstract] OR ageing adult*[Title/Abstract])) AND ("last 10 years"[PDat] AND ( English[lang] OR German[lang] ) )
